# Stretching a Semiflexible Polymer in a Tube

**DOI:** 10.3390/polym8090328

**Published:** 2016-09-09

**Authors:** Runhua Li, Jizeng Wang

**Affiliations:** Key Laboratory of Mechanics on Disaster and Environment in Western China, Ministry of Education, College of Civil Engineering and Mechanics, Lanzhou University, Lanzhou 730000, Gansu, China; lirh07@lzu.edu.cn

**Keywords:** wormlike chain model, tube confinement, Odijk length, stretch, GBR model, Brownian dynamics simulation

## Abstract

How the statistical behavior of semiflexible polymer chains may be affected by force stretching and tube confinement is a classical unsolved problem in polymer physics. Based on the Odijk deflection theory and normal mode decomposition in terms of Fourier expansion, we have derived a new compact formula for the extension of a wormlike chain of finite length strongly confined in a tube and simultaneously stretched by an external force. We have also suggested a new deflection length, which together with the force-extension relation is valid for a very extended range of the tube-diameter/persistence-length ratio comparing to the classic Odijk theory. The newly derived formula has no adjustable fitting parameters for the whole deflection regime; in contrast, the classic Odijk length needs different prefactors to fit the free energy and average extension, respectively. Brownian dynamics simulations based on the Generalized Bead-Rod (GBR) model were extensively performed, which justified the theoretical predictions.

## 1. Introduction

A variety of theoretical and technical applications [1,2,3,4,5,6,7] are subject to confinement and manipulation of macromolecules, which motivates researchers to explore physical interpretations of how the statistical behavior of polymer chains is affected when they are geometrically confined [8,9,10,11] and mechanically stretched [12,13]. For a sufficiently long polymer chain trapped in a cylindrical tube, studies based on wormlike chain (WLC) theory [14] have revealed various well-known regimes in terms of the ratio between tube diameter (*D*) and chain persistence length ($L_{p}$). When $D/L_{p}$ is very small, the confined polymer chain fluctuates back and forth against the tube wall, whose behaviors belong to the Odijk deflection regime, and the corresponding length scale is called the Odijk deflection length [8], $\lambda \sim D^{2/3}L_{p}^{1/3}$. However, quantitative applications of the Odijk length usually need a prefactor. Chen [15,16] has most recently found that such a prefactor, determined by fitting numerical predictions on free-energy, *F*, and average extension, $R_{\left|\right|}$, can be very different. As derived by Odijk [8,17], both *F* and $R_{\left|\right|}$ can be related to λ as

(1) $$F\approx \frac{L}{\lambda }k_{B}T$$

and

(2) $$1-\frac{R_{\left|\right|}}{L}\approx \frac{\vartheta \lambda }{2L_{p}}$$

where ϑ is a dimensionless proportionality constant. On the other hand, tube-confined WLCs in the Odijk regime have also been studied by using Monte Carlo and other methods by Burkhardt [18], Wang and Gao [19], Burkhardt et al. [20], Yang et al. [21] and Chen [16]. By assuming that configurations with “hairpins” [22] and excluded-volume effects are negligible, Yang et al. [21] and Chen [16] have determined the quantitative expression of the free energy of the chain with contour length *L* to be

(3) $$F=\frac{A_{O}R_{\left|\right|}}{L_{p}^{1/3}D^{2/3}}k_{B}T$$

and the extension $R_{\left|\right|}$ to be

(4) $$1-\frac{R_{\left|\right|}}{L}\approx \alpha_{O}{\left(\frac{D}{L_{p}}\right)}^{2/3}$$

respectively, where $A_{O}=2.3565$ and $\alpha_{O}=0.1701$. Comparing Equations (1)–(4), we can determine

(5) $$\lambda =A_{O}^{-1}L_{p}^{1/3}D^{2/3}$$

and the value $\vartheta =2\alpha_{O}A_{O}\approx 0.8015$.

Similarly, when a polymer chain is stretched by a force, the deflection regime still exists [17]. When the stretching force $f>>f_{cr}$, where $f_{cr}=k_{B}T/L_{p}$, the behavior of the polymer chain is in the deflection regime with Odijk length [17], $\lambda \approx L_{p}/\sqrt{\hat{f}}$, where $\hat{f}=fL_{p}/k_{B}T$. The extension of the chain can be related to λ as

(6) $$1-\frac{R_{\left|\right|}}{L}\approx \frac{1}{2\sqrt{\hat{f}}}$$

which has also been determined by Marko and Siggia [12] based on the technique of normal mode decomposition of an infinite WLC.

When the polymer chain simultaneously subjects to tube confinement and force stretch in the deflection regime, as shown by [23,24] and later by [25], the effect of confinement can be approximately equivalent to an additional effective stretching force $f_{c}$ [23] with

(7) $${\hat{f}}_{c}=c{\left(L_{p}/D\right)}^{4/3}$$

and ${\hat{f}}_{c}=f_{c}L_{p}/k_{B}T$, where *c* is a prefactor. Then force-extension relation of the confined polymer chain under stretching force, *f*, can be described by that of an unconfined chain subjecting to an effective stretching force *f* + *f*_c_. This has been verified and shown to be perfectly valid for long chains [26]. However, relevant expression on the effective force contains the adjustable parameter *c*, which depends on the tube dimension [23] and is totally different from $A_{O}$ and $\alpha_{O}$.

In spite of the above progress in the understanding of statistical behavior of confined polymers in the deflection regime, there are still open questions on how the Odijk length can be uniquely and precisely defined, so that this length scale can be valid for a very extended range of the ratio *D*/*L_p_*, and accordingly, for the behavior of a polymer chain with finite length and under the combined actions of tube confinement and force stretch, an accurate force-confinement-extension relation without adjustable parameters can be established. In this study, based on the Odijk deflection theory and an accurate expression on the confinement free energy for the chains with finite length by Yang et al. [21], we propose a modification to the classic deflection length scale. In terms of this modification, we derive the force-confinement-extension relation for the chain with finite length confined in a tube and stretched by an external force. We expect that the newly derived formula will be valid for both of the free energy and average extension without adjustable fitting parameters. Brownian dynamics simulations based on the Generalized Bead Rod (GBR) model [19] will be extensively performed to justify theoretical predictions.

## 2. Materials and Methods

### 2.1. Model

**Figure 1:**
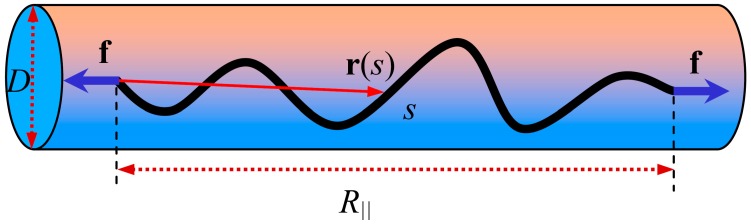
Schematic of a WLC confined in a tube and stretched by a force.

We use Figure 1 to show the model system of a WLC of contour length *L* strongly confined in a cylindrical tube with diameter *D*. We consider a set of Cartesian coordinates (*x*, *y*, *z*), which are placed at the center of the tube so that the *z* axis is along the tube axis, and unit vectors along the *x*, *y*, *z*-axis are **i**, **j** and **k**, respectively. The chain is stretched by a tensile force **f** = *f***k,** with *f* being a constant. The position and tangential vectors along the arc length *s* of the chain are

(8) $$r(s)=x(s)i+y(s)j+z(s)k=r_{⊥}\left(s\right)+z(s)k$$

(9) $$u(s)=\frac{dx}{ds}i+\frac{dy}{ds}j+\frac{dz}{ds}k=u_{⊥}+\frac{dz}{ds}k$$

where **r**(0) = **0**, and obviously we have

(10) $$r_{⊥}\left(s\right)=\int_{0}^{s}u_{⊥}\left(\xi \right)d\xi$$

Assuming small undulation, and noting inextensibility of the chain, we have ∥u∥=1 and $∥u_{⊥}∥<<1$. Further we can derive [23]

(11) $$\frac{\partial u}{\partial s}\approx \frac{\partial u_{⊥}}{\partial s}$$

The average extension or length of the tube occupied by the chain along *z*-axis becomes

(12) $$R_{∥}≜\langle z(L)-z(0)\rangle \approx L-\frac{1}{2}\int_{0}^{L}\langle u_{⊥}^{2}\rangle ds$$

The Hamiltonian of the confined WLC under stretch can be expressed as the summation of potential energies due to bending, stretching and tube-confinement as [8,12,13,23]

(13) $$H=\frac{L_{p}k_{B}T}{2}\int_{0}^{L}{\left(\frac{\partial u}{\partial s}\right)}^{2}ds-f\cdot [r(L)-r(0)]+\int_{0}^{L}V\left(r_{⊥}\right)ds$$

where

(14) $$V\left(r_{⊥}\right)=\{\begin{array}{cc} 0, & ∥r_{⊥}∥<D/2\\ \infty & \mathrm{otherwise} \end{array}$$

is the confinement potential per unit length due to the tube-wall [23]. Harmonic potential is usually adopted to approximate such a hard-wall potential [23,27], i.e.,

(15) $$V\left(r_{⊥}\right)\approx \frac{1}{2}\Xi r_{⊥}^{2}$$

in which Ξ can be viewed as a spring constant per unit length to be determined. Inserting Equations (8)–(11) and (15) into Equation (13) yields [23]

(16) $$H\approx \frac{L_{p}k_{B}T}{2}\int_{0}^{L}{\left(\frac{\partial u_{⊥}}{\partial s}\right)}^{2}ds+\frac{f}{2}\int_{0}^{L}u_{⊥}^{2}ds+\frac{\Xi }{2}\int_{0}^{L}{\left[\int_{0}^{s}u_{⊥}\left(\xi \right)d\xi \right]}^{2}ds$$

where a constant term has been dropped. We introduce the Fourier series expansion of $u_{⊥}\left(s\right)$ as

(17) $$u_{⊥}\left(s\right)=\sum_{n=-\infty }^{+\infty }u_{⊥n}\exp\left(\frac{2\pi ns}{L}i\right)$$

Substituting Equation (17) into Equation (16), we have

(18) $$H=\sum_{n=-\infty }^{+\infty }H_{n}$$

where

(19) $$\frac{H_{n}}{k_{B}T}=\left(\frac{\pi^{2}n^{2}}{l}+\hat{f}l+\frac{{\hat{f}}_{c}^{2}l^{3}}{4n^{2}\pi^{2}}\right)u_{⊥n}^{2}$$

and $\hat{f}=fL_{p}/k_{B}T$, ${\hat{f}}_{c}=2\sqrt{\Xi L_{p}^{3}/k_{B}T}$, and $l=L/2L_{p}$.

According to the equipartition theorem, $\langle H_{n}\rangle$ should be equal to $k_{B}T$ for two degrees of freedom, then from Equation (19) we can obtain the average undulation for each normal mode as

(20) $$\langle u_{⊥n}^{2}\rangle =1/\left(\frac{\pi^{2}n^{2}}{l}+\hat{f}l+\frac{{\hat{f}}_{c}^{2}l^{3}}{4n^{2}\pi^{2}}\right)$$

On the other hand, Equation (12) can be rewritten as

(21) $$R_{∥}\approx L-\frac{L}{2}\sum_{n=-\infty }^{+\infty }\langle u_{⊥n}^{2}\rangle$$

Inserting Equation (20) into Equation (21) yields

(22) $$1-\frac{R_{∥}}{L}\approx \frac{\varepsilon }{2\sqrt{\hat{f}+{\hat{f}}_{c}}}$$

where

(23) $$\varepsilon =\frac{f_{1}\cot\left(f_{1}l\right)-f_{2}\cot(f_{2}l)}{\sqrt{\hat{f}-{\hat{f}}_{c}}}$$

(24) $$f_{1}=\sqrt{-\hat{f}-\sqrt{{\hat{f}}^{2}-{\hat{f}}_{c}^{2}}}/\sqrt{2}$$

(25) $$f_{2}=\sqrt{-\hat{f}+\sqrt{{\hat{f}}^{2}-{\hat{f}}_{c}^{2}}}/\sqrt{2}$$

**Figure 2:**
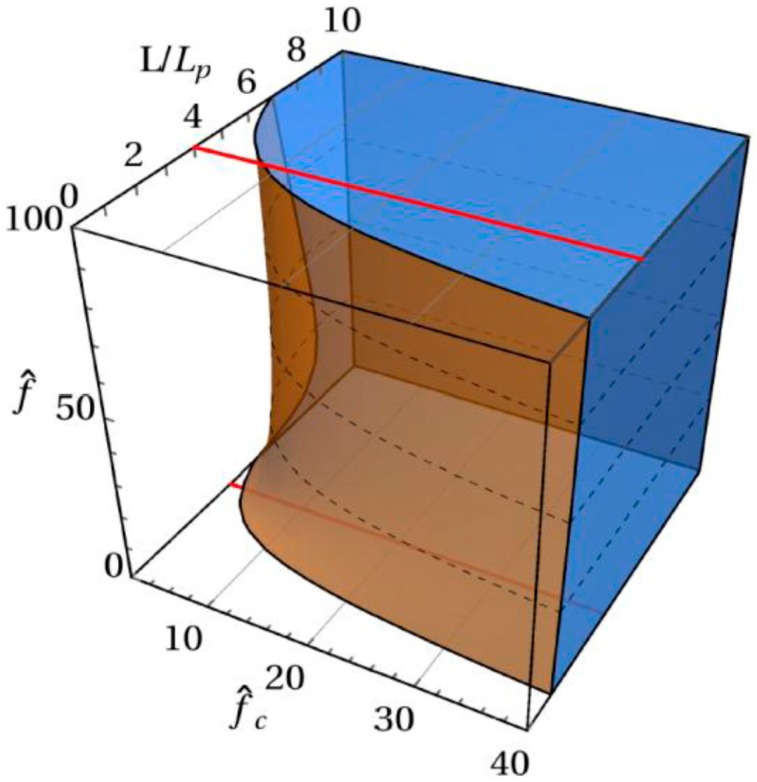
Dependence of the range 0.98<ε<1.02 on $\hat{f}$, ${\hat{f}}_{c}$, and $L/L_{p}$.

We note that an equivalent expression to Equations (22)–(25) has been derived by Burkhardt [25] by using a different method. Figure 2 shows the dependence of the range 0.98<ε<1.02 (shadow region) on $\hat{f}$, ${\hat{f}}_{c}$, and $L/L_{p}$, from which we can see that as long as the chain is sufficiently long and the confinement is strong enough, we always have ε≈1, so that Equation (22) can be simplified to

(26) $$1-\frac{R_{∥}}{L}\approx \frac{1}{2\sqrt{\hat{f}+{\hat{f}}_{c}}}$$

Actually, for an infinite long chain, Wang and Gao (2007) [23] have reached Equation (26) based on the technique of continuous Fourier transform. Recently, based on the same model, but a different method, a detailed study for general boundary conditions has been given by Burkhardt [24], in which Equation (26) is derived once again. From Equation (26), we can conclude that the effects of stretch and confinement of the chain can be decoupled under the condition of sufficiently long chain and strong confinement. The effect of confinement can be quantitatively represented by the effective force, ${\hat{f}}_{c}$, which is similar to what was concluded in [23] for the limiting case of an infinite long chain, in which the expression on the effective stretching force contains an adjustable parameter depending on the tube dimensions.

### 2.2. Modified Odijk Length and Effective Stretching Force Due to Confinement

When the WLC is confined in a tube or stretched by a force, as long as configurations with “hairpins” [22] are negligible, statistical behavior of the chain falls into the deflection regime. According to the Odijk deflection theory [8,17], we assume that the chain in the whole deflection regime can be considered as consisting of *L*/*λ* effective free segments so that the conformational free energy can still be scaled as Equation (1). On the other hand, when considering the average extension of the chain, each such segment behaves like a free chain with effective contour length $c_{1}\lambda$, where parameter *c*_1_ is introduced due to end effect of each segment. In addition, correlation of the tangent vector of an effective free segment is [28]

(27) $$\langle u\left(s_{1}\right)\cdot u\left(s_{2}\right)\rangle =e^{-\left|s_{2}-s_{1}\right|/L_{p}}$$

The extension of such an effective free segment,$R_{||\lambda }$, can be obtained by integrating Equation (27) as the form

(28) $$R_{||\lambda }=c_{2}\int_{s_{1}}^{s_{1}+c_{1}\lambda }\langle u\left(s_{1}\right)\cdot u\left(s_{2}\right)\rangle ds_{2}=c_{2}L_{p}(1-e^{c_{1}\lambda /L_{p}})$$

where $c_{1}$ and $c_{2}$ are unknown dimensionless factors. The average extension of the whole chain can be related to $R_{||\lambda }$ by

(29) $$R_{\left|\right|}=\frac{L}{\vartheta \lambda }R_{||\lambda }=c_{2}\frac{L_{p}L}{\vartheta \lambda }(1-e^{c_{1}\lambda /L_{p}})$$

Equation (29) should reproduce Equation (4) in the Odijk regime $D<<L_{p}<<L$, which can determine $c_{1}=\vartheta$ and $c_{2}=1$, so that eventually we obtain

(30) $$R_{\left|\right|}=\frac{L}{\vartheta \lambda }L_{p}\left(1-e^{-\vartheta \lambda /L_{p}}\right)$$

In many studies [15], researchers agree that the classic Odijk length in describing *F* and $R_{\left|\right|}$ has the same scaling law, ${\left(L_{p}D^{2}\right)}^{1/3}$, but different prefactors. How these prefactors are related is an unsolved problem in polymer physics. In this study, we assume that there exists only one deflection length, which satisfies both Equations (1) and (30) simultaneously. Eliminating *L* from these two equations, we obtain:

(31) $$\lambda =-\frac{L_{p}}{\vartheta }\ln(1-\frac{\vartheta R_{\left|\right|}k_{B}T}{FL_{p}})$$

Equation (31) can be regarded as a new deflection length that fulfills both requirements for the free energy and statistics of geometrical quantities.

Inserting Equation (3) into Equation (31), the new deflection length can be given by

(32) $$\lambda =-\frac{L_{p}}{\vartheta }\ln[1-\frac{\vartheta }{A_{O}}\left(\frac{D}{L_{p}}\right){}^{2/3}]$$

When $D/L_{p}$ <<1, Taylor expansion of Equation (32) yields Equation (5). On the other hand, when $\lambda /L_{p}$ is small, by performing Taylor expansion, Equation (30) can be approximated as

(33) $$1-\frac{R_{\left|\right|}}{L}\approx \frac{\vartheta \lambda }{2L_{p}}$$

**Figure 3:**
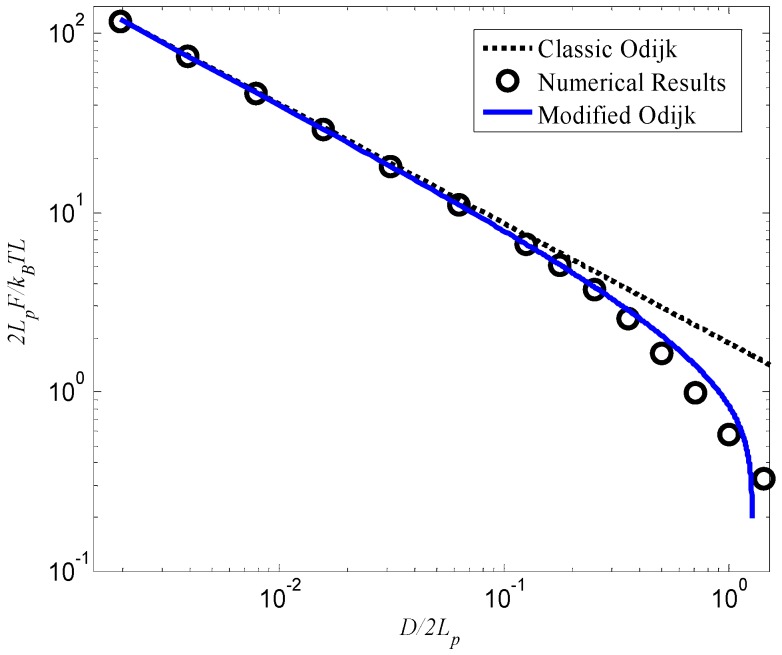
Normalized free energy as a function of the relative confinement diameter *D*/2*L_p_* for the circular confinement problem.

For the tube-confined WLC, Chen [16] derived a partial differential equation on the partition function of the system and numerically determined the free energy of the system by numerically finding the eigenvalue (EV) of an operator. Figure 3 shows the comparison between Chen’s numerical results, theoretical predictions based on the classic Odijk length and the modified Odijk length of Equation (32) in terms of Equation (1), respectively. It can be seen from Figure 3 that prediction based on the modified Odijk length can better match Chen’s results in [16], especially when *D*/*L_p_* becomes large.

We go back to the stretch of tube-confined WLC, and consider the average extension of the chain in Equation (26). When the applied force *f* = 0, Equation (26) becomes

(34) $$1-\frac{R_{∥}}{L}\approx \frac{1}{2\sqrt{{\hat{f}}_{c}}}$$

Equation (34) should be identical to Equation (33), so that we can derive the effective stretching force due to tube confinement as

(35) $${\hat{f}}_{c}\approx \frac{L_{p}^{2}}{\vartheta^{2}\lambda^{2}}$$

Substituting Equation (35) into Equation (26), and using the modified Odijk length, Equation (32), we obtain an expression without adjustable fitting parameters on the average extension of tube-confined WLC under stretch

(36) $$1-\frac{R_{\left|\right|}}{L}\approx \frac{1}{2\sqrt{\hat{f}+1/\ln^{2}\left[1-\frac{\vartheta }{A_{O}}{\left(\frac{D}{L_{p}}\right)}^{2/3}\right]}}$$

We note that Equation (36) is expected to be valid for a much extended range of *D/L_p_*. Only when *D/L_p_* << 1, Equation (36) can be reduced to

(37) $$1-\frac{R_{\left|\right|}}{L}\approx \frac{1}{2\sqrt{\hat{f}+\frac{1}{4\alpha_{O}^{2}}{\left(\frac{D}{L_{p}}\right)}^{-4/3}}}$$

### 2.3. Brownian Dynamics Simulations

We use Brownian dynamics simulations to verify the average extension expression of Equation (36) for the stretch of tube-confined WLCs. The simulations were performed based on the GBR model for the Brownian dynamics of strongly confined WLCs [19]. In the GBR model, a polymer chain is described as *N* identical virtual beads of radius, *a*, at positions, $r_{j}=\left(x_{j},y_{j},z_{j}\right)$, *j* = 1, 2, …, *N*, connected by *N* − 1 inextensible rods of length *b*. Once the position vector of *N* beads at time step *n*, $r_{\left(n\right)}$ (3*N* vector), is obtained, then the new position vector $r_{\left(n+1\right)}$ can be determined from [9,23,19]

(38) $$r_{\left(n+1\right)}=\left(I-T_{\left(n\right)}B_{\left(n\right)}\right)\left(r_{\left(n\right)}+\chi_{\left(n\right)}^{\mathrm{wall}}+\frac{\Delta t}{k_{B}T}D_{\left(n\right)}F_{\left(n\right)}+\xi_{\left(n\right)}\right)+T_{\left(n\right)}d$$

where $F_{\left(n\right)}$ is the collective vector of internal and external forces, $\xi_{\left(n\right)}$ is the vector of random force generated at each time step from a Gaussian distribution with zero mean and variance equal to

(39) $$\langle \xi_{\left(n\right)}\xi_{\left(n\prime \right)}\rangle =2D_{\left(n\right)}\Delta t\delta_{nn\prime }$$

and Δt is the time step, $\delta_{nn\prime }$ is the Kronecker delta symbol, $I-T_{\left(n\right)}B_{\left(n\right)}$ is a projection matrix which together with $T_{\left(n\right)}d$ sets the constraints, $\chi_{\left(n\right)}^{\mathrm{wall}}$ is the penalty displacement vector to realize tube confinement, and $D_{\left(n\right)}$ is the translational diffusion matrix determined through hydrodynamic interactions between beads.

Based on the GBR model, Brownian dynamics simulations have been performed for WLCs in nanotubes of different radii. In all simulations, the chains are initially set in a straight configuration. Tube confinements and constant tensile forces are then applied during the chains’ relaxation. Total simulation time for each single trajectory is 0.55 ms. We record the normalized end-to-end distance <*z*> of a chain along *z*-axis at each time increment. Each data point in the figures is obtained by averaging the recorded values of <*z*> for 16 trajectories with different random seeds, which is then denoted as $R_{∥}$. For all these Brownian dynamics simulations, we choose *L_p_* = 50 nm, viscosity of water $\eta_{0}=8.904\times 10^{-4}\;\mathrm{Pa}\cdot s$, and temperature T=298 K.

**Figure 4:**
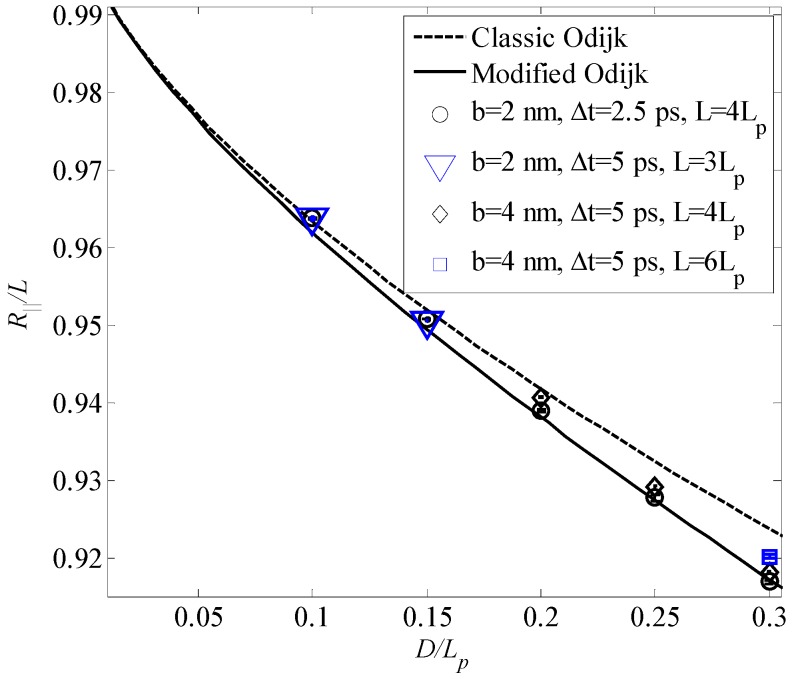
Average extension of the confined WLC without stretching as a function of the ratio *D*/*L_p_.*

Figure 4 shows the comparison of Brownian dynamics simulation results and corresponding theoretical predictions based on the classic Odijk length, $\lambda \approx \alpha_{O}D^{2/3}L_{p}{}^{1/3}$, and the modified Odijk length in Equation (32), for the normalized average extension of the WLCs confined in tubes of different diameters without stretching. Simulation parameters for bead radius, *a*, bond length, *b*, time step, Δt, and contour length, *L*, are *a* = 0.98, 0.98, 1.7 nm, *b* = 2, 2, 4 nm, Δt = 2.5 ps, 5 ps, 5 ps, and *L* = 4*L_p_*, 3*L_p_*, 4*L_p_*, 6*L_p_*, respectively. It can be seen from Figure 4 that results based on the newly derived formula for the average extension of the confined WLC agree with the simulation results well, and those based on the classic Odijk length shows discrepancy with the simulation results when the tube diameter becomes large.

**Figure 5:**
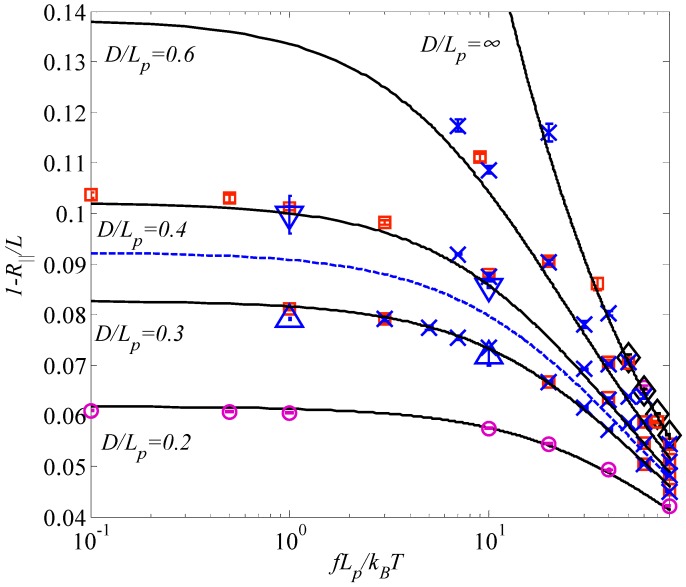
Comparison of Brownian dynamics simulation results and theoretical predictions on the relative average extension of the WLC confined in a tube and under stretch.

Figure 5 shows the comparison of Brownian dynamics simulation results and theoretical predictions based on Equations (36) and (37) associated with the modified and classic Odijk lengths, respectively, for the relative extension of nanotube-confined WLC under stretch. The simulations were conducted for chains of contour length *L* = 4*L_p_* and 6*L_p_* within different tubes under parameters *D*/*L_p_* = 0.2, 0.3, 0.4, 0.6, and ∞. Bead radius *a* = 0.98 nm corresponds to bond length *b* = 2 nm, whereas *a* = 1.7 nm corresponds to *b* = 4 nm. In Figure 5, the solid lines represent the predictions by using Equation (36), the dashed line represent the predictions by Equation (37) for *D*/*L_p_* = 0.4. Simulation parameters for the hollow circles are *b* = 2 nm, Δt = 2.5 ps, *L* = 4*L_p_*; X marks, *b* = 4 nm, Δt = 10 ps, *L* = 4*L_p_*; hollow squares, *b* = 4 nm, Δt = 5 ps; hollow diamonds, *b* = 2 nm, Δt = 5 ps, *L* = 4*L_p_*; hollow triangles (up), *b* = 4 nm, Δt = 5 ps, *L* = 6*L_p_*; hollow triangles (down), *b* = 4 nm, Δt = 5 ps, *L* = 6*L_p_*. It can be seen from Figure 5 that the theoretical predictions by Equation (36) based on the modified Odijk length agree with Brownian dynamics simulation results very well when $R_{∥}/L<0.9$. Most importantly, no fitting parameters are used in these comparisons. A large discrepancy exists between the simulation results and the predictions using Equation (37) based on the classic Odijk length for *D*/*L_p_* = 0.4.

## 3. Discussion

We have theoretically and numerically studied the average extensions of tube-confined semiflexible polymer chains under stretch in the deflection regime. We derived a new deflection length which unified the concept of Odijk length in free energy and geometry understandings, respectively, and accordingly we obtained a compact formula on the force-confinement-extension relation without any adjustable fitting parameters. In terms of Brownian dynamics simulations, these theoretical predictions have been confirmed to be valid for a much more extended range of the ratio, *D*/*L_p_*. On the other hand, our prediction on the effective stretching force due to the tube confinement has been justified to be valid for $R_{∥}/L<0.9$ and $\hat{f}<100$. We thus hypothesis that, no matter the WLCs are confined in a tube and/or stretched by a force, they are in the deflection regime as long as $R_{∥}/L<0.9$.

## 4. Conclusions

In conclusion, for the statistical behavior of semiflexible polymers simultaneously subject to force stretching and tube confinement, we have redefined and derived a new deflection length scale, which has been numerically justified to be valid in the regime beyond that of the classic Odijk. Based on such a new length scale, we have further derived a formula without open parameters to successfully describe the force-stretching relation of tube-confined semiflexible polymers. This study solves the dilemma for the classic Odijk length that which prefactors should be chosen in describing the statistical behaviors of polymers under complex microenvironments other than only geometrical confinements.
